# Impact of residual tumor cells in the stem cell collection on multiple myeloma patients receiving autologous stem cell transplantation

**DOI:** 10.1007/s00277-023-05427-8

**Published:** 2023-09-08

**Authors:** Jingyu Xu, Wenqiang Yan, Huishou Fan, Jiahui Liu, Lingna Li, Chenxing Du, Shuhui Deng, Weiwei Sui, Yan Xu, Lugui Qiu, Gang An

**Affiliations:** 1 State Key Laboratory of Experimental Hematology, National Clinical Research Center for Blood Diseases, Haihe Laboratory of Cell Ecosystem, Institute of Hematology & Blood Diseases Hospital, Chinese Academy of Medical Sciences & Peking Union Medical College, 288 Nanjing Road, Tianjin, 300020 China; 2 Tianjin Institutes of Health Science, Tianjin, 301600 China

**Keywords:** Autologous stem cell transplantation, Minimal residual disease, Multiple myeloma, Stem cell collection, Prognosis

## Abstract

Autologous stem cell transplantation (ASCT) is the standard therapy for patients with transplant-eligible multiple myeloma (TEMM). However, the ideal depth of response required before ASCT and the impact of residual tumor cells in the stem cell collection (SCC) on survival remains unclear. Here we collected data of 89 patients with TEMM undergoing ASCT and analyzed the minimal residual disease of SCC (cMRD) and bone marrow (BM) (mMRD) before transplantation. Before ASCT, 31.5% and 76.4% of patients achieved MRD negativity in BM and SCC, respectively. Tumor cells were less in SCC samples than that in BM samples. Neoplastic cells in SCC could be observed in patients with different responses after induction therapy, and there were no significant differences in the percentage and level of cMRD among these subgroups (*P* > 0.05). No correlation was found between the cMRD status and the response patients achieved after ASCT (*P* > 0.05). The median follow-up was 26.8 months. mMRD negativity before ASCT was associated with longer PFS (55.9 vs. 27.1 months; *P* = 0.009) but not OS (not reached vs. 58.9 months; *P* = 0.115). Patients with different cMRD statuses before ASCT experienced similar PFS (40.5 vs. 76.4 months for negativity vs. positivity; *P* = 0.685) and OS (not reached vs. 58.8 months for negativity vs. positivity; *P* = 0.889). These results suggested that detectable cMRD does not significantly predict the inferior post-ASCT response or shorter survival, and patients are eligible to undergo ASCT upon achieving partial response.

**Supplementary Information:**

The online version contains supplementary material available at 10.1007/s00277-023-05427-8.

## Introduction

Multiple myeloma (MM) is the second most common hematological malignancy associated with monoclonal plasma cells (PCs). In recent years, novel treatment options for MM have become widely available, leading to substantial improvement in response and prognosis [1]. The IFM 2013-04 trial [2] was the first to verify the significant and synergistic activity of a proteasome inhibitor (PI) combined with an immunomodulatory drug (IMiD), and the prospective randomized phase 3 trial SWOG S0777 [3] has demonstrated the superior efficacy and survival benefit of the VRd regimen (bortezomib, lenalidomide, and dexamethasone), and the combination regimen has been incorporated into front-line induction therapy for MM [4, 5]. Although these novel drugs have the potential to significantly improve patient survival, several studies have highlighted the irreplaceable role of autologous stem cell transplantation (ASCT) in transplant-eligible patients with MM (TEMM) [6, 7].

Most clinical guidelines recommend ASCT for patients who have achieved partial response (PR), and previous studies [8, 9] also revealed that prolonging the induction therapy duration or additional pre-ASCT salvage chemotherapy could deepen the depth of the pre-ASCT response but it was not associated with survival benefits. However, whether it would result in the presence of tumor cells within the stem cell collection (SCC) in patients failing to achieve complete response (CR) and its impact on patient outcome remain to be elucidated. Some studies have focused on the prognostic value of the MRD of SCC (cMRD) prior to the transplantation, but no consensus has been reached [10, 11].

In this study, we aimed to evaluate the effect of minimal residual disease (MRD) in patients with TEMM by comparing the number of tumor cells in SCC and bone marrow (BM) samples obtained before ASCT. We also aimed to investigate the effect of MRD in SCC samples on treatment responses and survival.

## Materials and methods

### Patients

We analyzed the clinical data of 89 patients with MM who underwent ASCT at our hospital between January 1, 2013, and June 1, 2021. All patients were included in a prospective, non-randomized clinical trial (BDH 2008/02), and the treatment regimen has been described in previous studies [12, 13].

The enrolled patients in our data all received PIs-based induction therapy. After attaining PR at minimum after at least four cycles, patients underwent ASCT followed by maintenance. All patients underwent melphalan treatment after induction with granulocyte colony-stimulating factor (G-CSF) administered for mobilization. Plerixafor was used in cases of suboptimal mobilization and collection. The minimum CD34^+^ stem cell dose for collection was 2 × 10^6^ CD34^+^ cells/kg, and the optimal target was 4 × 10^6^ CD34^+^ cells/kg. Responses were assessed in accordance with International Myeloma Working Group (IMWG) criteria [14]. In our study, the best response was defined as the deepest response during follow-up. Time from ASCT to disease progression was defined as progression-free survival (PFS) and time from ASCT to death as overall survival (OS).

### Fluorescence in situ hybridization and flow cytometry analysis

CD138^+^ PC enrichment and fluorescence in situ hybridization (FISH) analyses were performed as previously described [15]. The routine panel included evaluations for 13q14 deletion, 17p deletion, 1q21 gain/amplification, t(11;14), t(4;14), t(14;16), and t(14;20). Positive cutoff levels were defined as 10% for translocation and 20% for numerical abnormalities [16]. High-risk cytogenetic abnormalities (CAs) were defined as the presence of del(17p), t(4;14), or t(14;16) [17]. MRD was evaluated via multiparameter flow cytometry with a sensitivity of 1 × 10^−5^ using two combinations of 8-color monoclonal antibodies as previously described [18].

### Statistical analysis

Two-sided chi-square tests and Fisher’s exact tests were used to assess differences between categorical variables. Wilcoxon tests and Mann–Whitney *U* tests were used to assess differences between continuous variables. Survival outcomes were analyzed using the Kaplan–Meier method and compared using the log-rank test. The Cox proportional hazard model was used to estimate hazard ratios (HRs) and 95% confidence intervals (CIs). Statistical analyses were performed using SPSS version 26 (IBM, Armonk, NY, USA) and R Studio version 4.1.2. *P* < 0.05 was considered statistically significant. For significant values in univariate analysis (*P* < 0.10), the Cox proportional hazards model was used to identify independent predictive factors.

## Results

### Patient characteristics

A total of 89 patients were included in the analysis. The baseline characteristics are presented in Supplementary Table S1. The median age of the patients was 54 years (range, 37–69 years), and 62.9% were male. FISH identified high-risk CAs in 24 patients (27.0%). Before ASCT, the percentages of patients with mMRD− and cMRD− were 31.5% and 76.4%, respectively. The baseline characteristics of patients with different MRD statuses in SCC and BM were similar (Table 1).

**Table 1:** Baseline characteristics and responses of patients according to MRD status in the SCC and BM

Characteristics	cMRD− (*n* = 68)	cMRD+ (*n* = 21)	*P* value	mMRD− (*n* = 28)	mMRD+ (*n* = 61)	*P* value
Age (years)	54 [39–69]	55 [37–69]	0.526	54 [41–64]	54 [37–69]	0.274
Sex			0.684			0.444
Male	42/68 (61.8)	14/21 (66.7)		16/28 (57.1)	40/61 (65.6)	
Female	26/68 (38.2)	7/21 (33.3)		12/28 (42.9)	21/59 (34.3)	
Immunoglobulin subtype			0.346			0.130
IgG	35/68 (52.2)	7/21 (33.3)		11/28 (39.3)	29/59 (49.2)	
IgA	20/68 (29.0)	6/21 (28.7)		9/28 (32.1)	18/59 (30.5)	
Light chain	6/68 (8.7)	4/21 (19.0)		5/28 (17.9)	5/59 (8.5)	
IgD	3/68 (4.3)	2/21 (9.5)		3/28 (10.7)	2/59 (3.4)	
Non-secretory	4/68 (5.8)	2/21 (9.5)		0 (0)	5/59 (8.5)	
DS stage			0.159			1.000
I	1/67 (1.5)	0 (0)		0 (0)	1/61 (1.6)	
II	4/67 (6.0)	4/21 (19.0)		2/27 (7.4)	6/61 (9.8)	
III	62/67 (92.5)	17/21 (81.0)		25/27 (92.6)	54/61 (88.5)	
ISS stage			0.423			0.369
I	8/64 (12.5)	3/21 (14.3)		5/26 (19.2)	6/59 (10.2)	
II	34/64 (53.1)	8/21 (38.1)		10/26 (38.5)	32/59 (54.2)	
III	22/64 (34.4)	10/21 (47.6)		11/26 (42.3)	21/59 (35.6)	
R-ISS stage			0.104			0.693
I	4/62 (6.5)	3/21 (14.3)		3/24 (12.5)	4/59 (6.8)	
II	45/62 (72.5)	10/21 (47.6)		15/24 (62.5)	40/59 (67.8)	
III	13/62 (21.0)	8/21 (38.1)		6/24 (25.0)	15/59 (25.4)	
Cytogenetic risk			0.509			0.678
Standard risk	36/55 (65.5)	14/19 (73.7)		17/24 (70.8)	32/48 (66.7)	
High risk	19/55 (34.5)	5/19 (26.3)		9/24 (29.2)	36/48 (33.3)	
Laboratory values at diagnosis						
Hb (g/L)	94.5 [55–133]	96 [72–162]	0.883	97 [66–154]	95 [55–162]	0.732
Serum albumin (g/L)	34.2 [18.5–47.9]	34.7 [23–49.2]	0.636	37.9 [19.1–47.9]	31.7 [18.5–49.2]	0.047
Serum β2-MG (mg/L)	4.44 [1.57–44.1]	4.33 [2.20–18.3]	0.888	4.93 [1.57–44.1]	4.33 [1.57–18.3]	0.643
Serum creatinine (μmol/L)	74.5 [43–490]	74.7 [45.8–450.3]	0.889	79.8 [43.0–363.8]	73.1 [45.8–490.8]	0.552
Serum calcium (mmol/L)	2.29 [1.86–4.01]	2.33 [1.93–3.01]	0.787	2.41 [1.86–4.01]	2.26 [1.88–3.01]	0.018
LDH	165 [46.5–620.3]	172.6 [75.9–304.6]	0.744	185.7 [66.5–620.3]	160.3 [46.5–331.0]	0.239
Plerixafor			0.912			0.770
Yes	25/68 (36.8)	8/21 (38.1)		11/31 (39.3)	22/59 (37.3)	
No	43/68 (63.2)	13/21 (61.9)		17/31 (60.7)	37/59 (62.7)	
Number of progression events after ASCT	23/68 (33.8)	6/21(28.6)	0.654	6/28 (21.4)	23/61 (37.7)	0.128
Number of deaths after ASCT	12/68 (17.6)	1/21 (4.8)	0.268	3/28 (10.7)	10/61 (16.4)	0.703

Values are presented as median [range] or *n*/*n* (%)

*MRD*, minimal residual disease; *SCC*, stem cell collection; *BM*, bone marrow; *cMRD*, minimal residual disease in the stem cell collection; *mMRD*, minimal residual disease in the bone marrow; *Ig*, immunoglobulin; *DS*, Durie-Salmon; *ISS*, International Staging System; *R-ISS*, Revised International Staging System; *Hb*, hemoglobin; *β2-MG*, β2-microglobulin; *LDH*, lactate dehydrogenase; *PFS*, progression-free survival; *ASCT*, autologous stem cell transplantation

### Tumor cells in the SCC and BM

Regardless of the sensitivity used for cMRD evaluation, the percentage of patients with MRD positivity in the BM was much higher than that with positivity in the SCC (69.6% vs. 24.4% for the sensitivity of 10^−6^, 68.5% vs. 23.3% for the sensitivity of 10^−5^, and 57.3% vs. 12.2% for the sensitivity of 10^−4^; *P* < 0.001; Supplementary Table S2). As for the numeric level of tumor cells in BM and SCC samples, the distribution of the detectable monoclonal PCs was 48.4% vs. 77.3% for the 10^1^–10^2^ level and 51.6% vs. 22.7% for the 10^3^–10^5^ level (*P* = 0.019; Supplementary Table S2).

Plerixafor, a CXC chemokine receptor 4 (CXCR4) antagonist, is widely used in clinical practice as a stem cell-mobilizing agent. CXCR4 is expressed not only on BM progenitor cells but also on various types of tumor cells [19, 20]. Supplementary Table S3 shows a comparison of cMRD status according to plerixafor use. Plerixafor was used during the mobilization of hematopoietic stem cells in 33 patients. Notably, the frequency of residual clonal PCs in SCC was similar between the plerixafor and non-plerixafor groups (24.2% vs. 23.2%; *P* = 0.912), and there was no significant difference in the median level of cMRD (0.008% with plerixafor and 0.01% without plerixafor; *P* = 0.920).

### Correlation between response and cMRD

The response of patients at stem cell collecting and after transplantation is shown in Table 2. Although the percentages of patients achieving at least very good PR (≥VGPR, 76.1% vs. 52.4%; *P* = 0.037) and CR (≥CR, 49.2% vs. 23.8%; *P* = 0.040) were higher among patients with cMRD− than those among patients with cMRD+ before collection, the rates of deep response after ASCT were similar between the cMRD+ and cMRD− groups (*P* > 0.1; Table 2), which was in contrast with the findings observed for patients with different mMRD statuses.

**Table 2:** Responses according to MRD status in the SCC

Response	All patients (n=89)	cMRD− (*n* = 68)	cMRD+ (*n* = 21)	*P* value
Response before collection				
≥VGPR	62/88 (70.4)	51/67 (76.1)	11/21 (52.4)	0.037
≥CR	38/88 (43.2)	33/67 (49.2)	5/21 (23.8)	0.040
Day 100 response after ASCT				
≥VGPR	67/87 (77.0)	55/67 (82.1)	13/20 (65.0)	0.189
≥CR	47/87 (54.0)	38/67 (56.7)	8/20 (40.0)	0.189
Best response after ASCT				
≥VGPR	74/83 (89.2)	60/65 (92.3)	14/18 (77.8)	0.185
≥CR	60/83 (72.3)	49/65 (75.4)	11/18 (61.1)	0.368

Values are presented as *n*/*n* (%)

*MRD*, minimal residual disease; *SCC*, stem cell collection; *BM*, bone marrow; *cMRD*, minimal residual disease in stem cell collection; *mMRD*, minimal residual disease in the bone marrow; *ASCT*, autologous stem cell transplantation; *VGPR*, very good partial response; *CR*, complete response

**Fig. 1:**
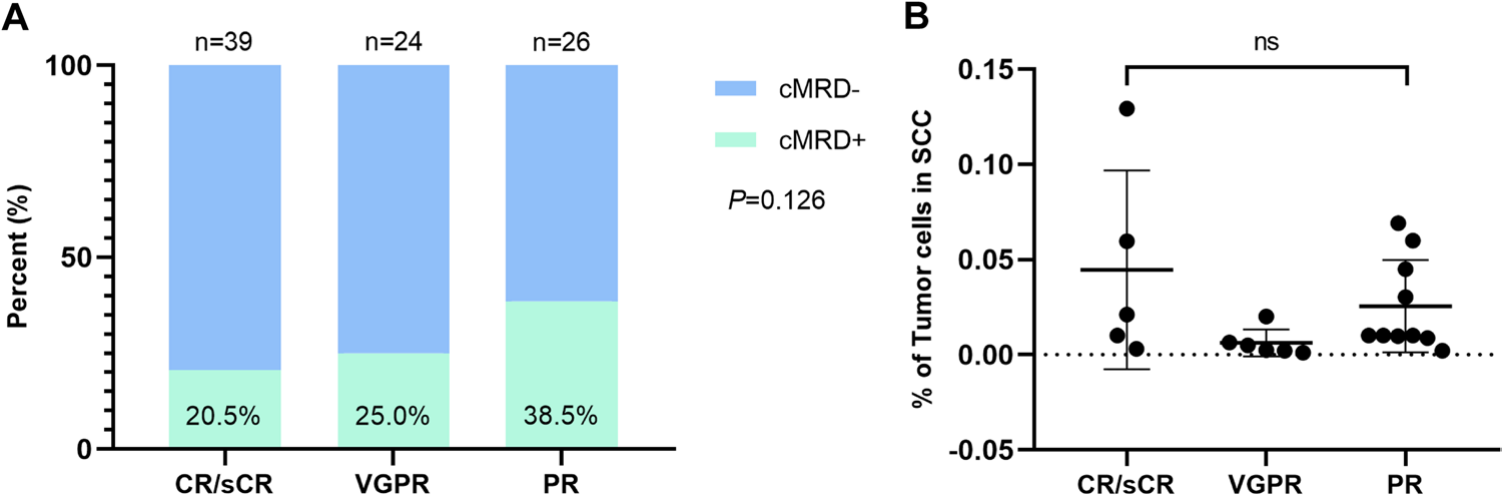
Presence (**A**) and level (**B**) of clonal plasma cells according to the response after induction therapy. MRD, minimal residual disease; SCC, stem cell collection; cMRD, minimal residual disease in stem cell collection; CR, complete response; sCR, stringent complete response; VGPR, very good partial response; PR, partial response

Residual tumor cells were observed in the SCC in 20.5% and 25.0% of patients who attained CR/sCR or VGPR after induction therapy, respectively. Although a higher percentage (38.5%) of cMRD+ was observed in patients with PR before ASCT, there were no significant differences among the three subgroups (*P* = 0.126; Fig. 1A). Similarly, we observed no significant differences in the level of cMRD among patients with PR, VGPR, and CR/sCR (*P* = 0.568; Fig. 1B). Because of the relatively higher percentage of cMRD+ in patients achieving PR, we then analyzed post-ASCT response of these patients. Approximately 29.9% (26/87) of the enrolled patients achieved PR after induction therapy, with 10 and 16 patients exhibiting cMRD+ and cMRD−, respectively, and both groups achieved similar deep response rate for post-ASCT response (40% vs. 43.7% for VGPR or better, 10% vs. 12.5% for CR or better) and the best response during the follow-up (60% vs. 66.6% for VGPR or better; 30% vs. 20% for CR or better, Supplementary Table S4).

### Prognostic value of post-induction and post-ASCT responses

**Fig. 2:**
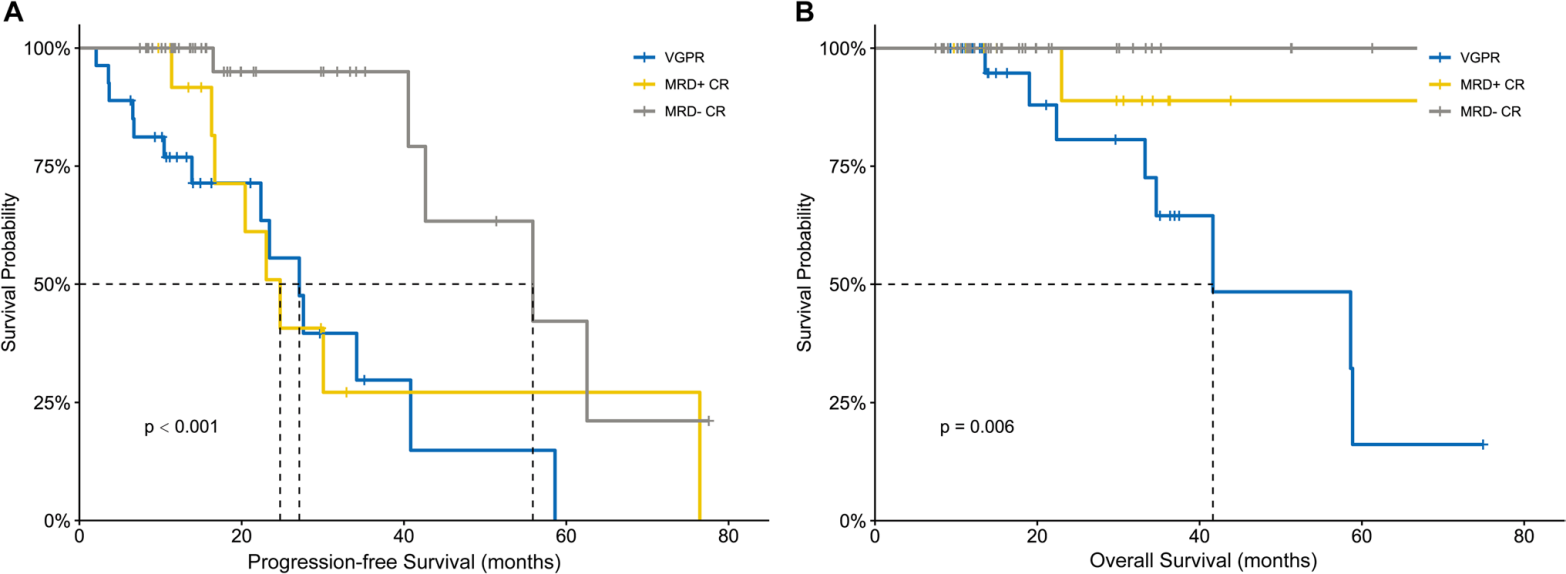
Survival curves according to the best response and MRD status after ASCT in all patients. PFS (**A**) and OS (**B**) in patients achieving ≤VGPR, MRD+ CR, and MRD− CR. MRD, minimal residual disease; ASCT, autologous stem cell transplantation; PFS, progression-free survival; OS, overall survival; VGPR, very good partial response; CR, complete response

The median duration of follow-up was 26.8 months (15.1–105.1 months). Supplementary Figure S1 shows the results of the survival analysis for patients with different responses before and after ASCT. Pre-ASCT response lacked a survival benefit for PFS (*P* = 0.195; Supplementary Figure S1A) and OS (*P* = 0.168; Supplementary Figure S1B). In contrast, post-ASCT response was identified as a significant predictor of survival. Better responses were associated with increase in both PFS (not reached vs. 42.6 vs. 27.1 vs. 30.1 for sCR vs. CR vs. VGPR vs. PR; *P* = 0.011; Supplementary Figure S1C) and OS (not reached vs. not reached vs. 58.6 vs. 41.6 for sCR vs. CR vs. VGPR vs. PR; *P* = 0.007; Supplementary Figure S1D). Similar results were obtained for best response and mMRD status during follow-up (Fig. 2), with median PFS of 27.2 months among patients with VGPR or less, 24.7 months among patients with mMRD+ CR, and 55.9 months among patients with mMRD− CR (*P* < 0.001; Fig. 2A). The median OS was 41.6 months in the VGPR or less group and not reached in the other two CR subgroups (*P* = 0.006; Fig. 2B).

### Prognostic value of cMRD and mMRD

**Fig. 3:**
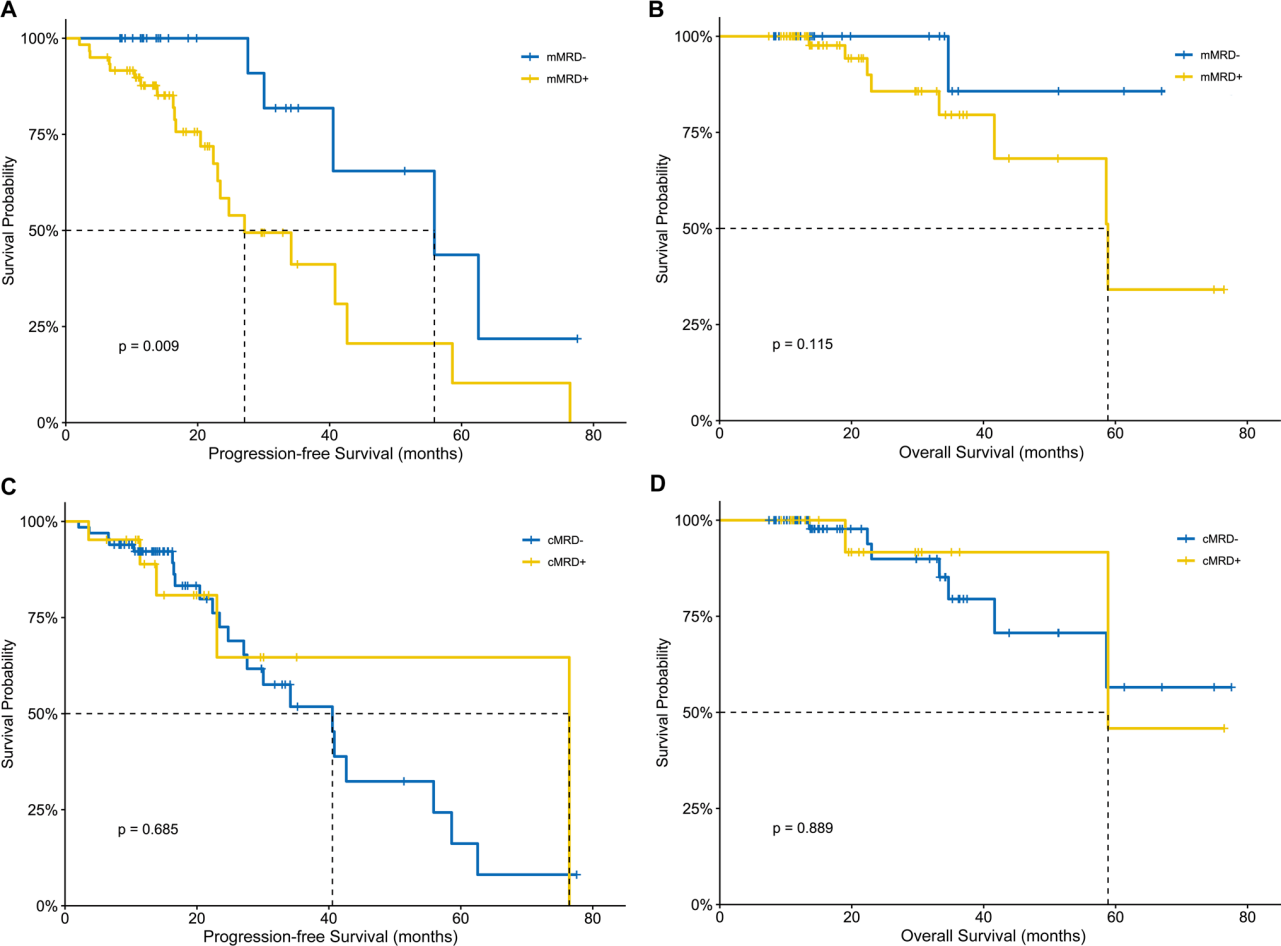
Kaplan–Meier survival analysis for patients with multiple myeloma according to MRD status in the BM (**A**, **B**) and SCC (**C**, **D**) before ASCT. MRD, minimal residual disease; SCC, stem cell collection; BM, bone marrow; cMRD, minimal residual disease in stem cell collection; mMRD, minimal residual disease in the bone marrow; ASCT, autologous stem cell transplantation

Negative mMRD before ASCT was associated with longer PFS (55.9 vs. 27.1 months; *P* = 0.009; Fig. 3A); however, no significant difference was observed in the OS according to pre-ASCT mMRD status (not reached vs. 58.9 months for mMRD− vs. mMRD+; *P* = 0.115; Fig. 3B). Median PFS (40.5 vs. 76.4 months for cMRD− vs. cMRD+; *P* = 0.685; Fig. 3C) and OS (not reached vs. 58.8 months for cMRD− vs. cMRD+; *P* = 0.889; Fig. 3D) did not significantly differ according to cMRD status before ASCT.

Multivariate Cox regression analysis was performed to examine associations among the variables sex, International Staging System (ISS) stage, cytogenetic risk, lactate dehydrogenase (LDH), mMRD, cMRD, response at day 100 after ASCT, and best response during follow-up. None of these factors demonstrated independent significance for OS (*P* ≥ 0.05). LDH level and the best response during follow-up remained significant prognostic factors for PFS. However, pre-ASCT cMRD exerted no influence on patient outcomes (Table 3).

**Table 3:** Cox proportional hazards models for PFS

Variables	PFS			
	Univariate analysis		Multivariate analysis	
	HR (95% CI)	*P* value	HR (95% CI)	*P* value
Male vs. female	1.12 (0.46–2.72)	0.800	NA	NA
ISS stage III vs. stage I/II	3.02 (0.88–10.45)	**0.080**	2.14 (0.57–8.03)	0.260
HR vs. SR	1.40 (0.58–3.38)	0.465	NA	NA
High vs. normal LDH	2.25 (0.87–5.82)	**0.094**	4.03 (1.47–11.09)	**0.007**
mMRD+ vs. mMRD− before ASCT	3.44 (1.28–9.23)	**0.014**	0.69 (0.15–3.17)	0.635
cMRD+ vs. cMRD− before ASCT	1.04 (0.41–2.61)	0.937	NA	NA
MRD− CR vs. ≤VGPR or MRD+^†^	0.22 (0.07–0.63)	**0.005**	0.29 (0.06–1.34)	0.112
MRD− CR vs. ≤VGPR^‡^	0.10 (0.03–0.34)	**<0.001**	0.12 (0.02–0.77)	**0.048**
MRD− CR vs. MRD+ CR^‡^	0.19 (0.05–0.63)	**0.007**	0.70 (0.25–1.98)	0.501

In the univariate analysis, significance was defined as *P* < 0.1

^†^Depth of response and MRD status on day 100 after ASCT

^‡^Best response and MRD status after ASCT

*PFS*, progression-free survival; *HR*, hazard ratio; *CI*, confidence interval; *ISS*, International Staging System; *HR*, high risk; *SR*, standard risk; *LDH*, lactate dehydrogenase; *cMRD*, minimal residual disease in the stem cell collection; *mMRD*, minimal residual disease in the bone marrow; *MRD*, minimal residual disease; *ASCT*, autologous stem cell transplantation; *VGPR*, very good partial response; *CR*, complete response

## Discussion

As a standard of care, ASCT has provided substantial benefit for patients with TEMM, resulting in a deeper response and survival improvement [7, 21, 22]. Sufficient evidence has demonstrated the significant prognostic value of a deep response and mMRD− following ASCT [23, 24]. Although many studies have investigated the clinical value of mMRD for predicting survival, relatively few have focused on cMRD before ASCT. Furthermore, studies evaluating the association between SCC contamination and outcomes have yielded contradictory results [10, 25, 26]. Whether it would lead to the presence of tumor cells in the collection when patients have not achieved CR and whether cMRD+ is associated with an increased risk of progression remains to be elucidated.

In this study, tumor cells were detected in BM in 68.5% of the patients, but only 23.6% of the patients exhibited tumor cells in the SCC, and the presence of latter was not correlated with the baseline characteristics of the cohort. Thereafter, we compared the tumor cells in the SCC and BM according to different sensitivities and numeric levels, which yielded similar results that the tumor cells were less common in the SCC than that in the BM. MM cells are drastically affected by the BM milieu for its dependence on the protection and immunosuppressive effect provided by the tumor microenvironment (TME), which favors tumor cell immune escape and drug resistance, thus promoting disease progression [20]. This may explain why there were more MM cells in the BM than in circulation. We also investigated whether MM cells could be mobilized into the circulation using plerixafor and found that the application of plerixafor did not increase the percentage of MRD-positive collections, while the level of neoplastic plasma cells in the collection in plerixafor group was higher than that in no-plerixafor group. A previous study reported a significant increase in the mobilization of MM cells from the BM to circulation, but the study was only in vivo without clinical data analysis [27]. This phenomenon was explained as a surrogate marker for the disruption of adhesion, and the authors suggested that plerixafor enhanced bortezomib-induced tumor reduction. Based on these results, we conclude that the addition of plerixafor to G-CSF treatment is a safe mobilizing regimen for patients with MM.

Our findings indicated that although patients with cMRD− obtained superior response prior to ASCT, the difference of response between patients with different cMRD statuses was not observed after the transplantation, demonstrating that graft contamination had no impact on ASCT. Besides, the inferior response did not increase the presence or level of tumor cells in the SCC. Patients who achieved PR before ASCT and those with VGPR or better response before ASCT both showed evidence of cMRD positivity. Evaluation of conventional response cannot accurately reflect the level of mMRD accurately even if patients have achieved CR. Therefore, it is highly probable that clonal PCs may still exist in the BM and mobilized into the peripheral blood.

As reported in previous studies [8, 9], our study revealed the same conclusion that the depth of the pre-ASCT response provided less survival benefit than the post-ASCT response. Vij et al. [8] reported that salvage therapy such as those with novel agent combinations and additional pre-ASCT salvage chemotherapy improved the depth of response but was not associated with survival benefits for patients achieving a suboptimal response (less than PR) to initial induction therapy. This is in contrast with the findings for acute leukemia, in which a deep response is necessary before transplantation. In our study, the post-ASCT and best responses were significantly associated with survival. These findings suggested that the post-ASCT response was a more important indicator of survival than the pre-ASCT response, which is consistent with other studies demonstrating the clinical value of a superior response after ASCT [23, 28].

The MRD, a new criterion for assessing treatment response introduced by the IMWG in 2016 [14], has been a hot topic for a few years. Undetectable MRD in the BM, especially sustained MRD negativity, is considered a more valuable prognostic marker for longer PFS and OS than CR [23, 29, 30]. Our results also implicated that for patients with post-ASCT CR, undetectable mMRD indicates better survival benefit compared with mMRD positivity. Furthermore, we found that mMRD status before ASCT can be considered a predictor of PFS, consistent with the findings of previous studies in our center. However, the correlation between pre-ASCT mMRD status and OS was not found statistically significant, but there was a stratified trend in survival curves, which may be related to the inclusion of limited data, the relatively short duration of follow-up and great prognosis of patients with ASCT. However, pre-ASCT mMRD negativity was not an independent prognostic factor for PFS in the COX multivariate analysis, which may be related to the short follow-up and positive impact of ASCT on tumor burden in BM [31].

Some studies have focused on the prognostic value of cMRD prior to the transplantation; however, no consensus has been reached. Our study demonstrated that the presence of tumor cells in the SCC had no effect on survival among patients with MM undergoing ASCT. We further verified these results by comparing cMRD status among patients with different responses before and after ASCT. This phenomenon may be related to the inactivation or death of MM cells in the frozen stock solution following apheresis due to their high dependence on the TME. A phase 3 clinical trial [10] in 2001 aimed on purging of autologous peripheral-blood stem cells using CD34 selection demonstrated that CD34 selection could significantly reduce myeloma cell contamination in SCC, but it did not reduce the risk of disease progression. Also, Boccadoro et al. [32] and Ho et al. [25] reported that the relevance was not found between tumor cells in the SCC and survival in their studies. However, some studies have yielded contradictory findings. In 1997, a study revealed that when monoclonal PCs in the blood stem cell harvest increase to 0.2 × 10^6^/L, it could predict a shortened relapse-free survival [33]. However, the number of patients enrolled in this research was only 33, and the patients did not undergo ASCT until progression occurred, also the tumor cells in the BM and the heavy tumor burden may have contributed to the disease progression as well. The study of Wuillème et al. [34] highlighted the impact of neoplastic PCs in the peripheral blood on the day of stem cell collection using flow cytometric detection, revealing that patients in MRD negativity group showed longer PFS and OS. However, the number of patients included in this study was only 75 and more patients with MRD negativity received the tandem transplantation.

Our study has some limitations. First, the limited cohort size may have had some impact on the analysis, and updated data are therefore required to verify our conclusions. Second, for the lack of the related data, there was no comparison between the MRD of collection and the MRD of graft. Last, this was a real-world retrospective study conducted at a single center and there may be selection bias for patients.

## Conclusion

Our results provide new insight suggesting that the level of tumor cells in the SCC of patients before transplantation is significantly less than that in the BM, and this study reveals the similar level of tumor cells in SCC for patients with different responses before ASCT. Detectable cMRD (with the sensitivity of 1 × 10^−5^) does not significantly predict the inferior post-ASCT response or shorter survival, highlighting the feasibility of ASCT in patients who have attained PR.

### Supplementary information

ESM 1 (DOCX 74.7 KB)

## Data Availability

The datasets generated during and/or analyzed during the current study are available from the corresponding author on reasonable request.
