# Supplementary material for: Impact of residual tumor cells in the stem cell collection on multiple myeloma patients receiving autologous stem cell transplantation
Source: Ann Hematol. 2023 Sep 8;102(11):3195–204. doi: 10.1007/s00277-023-05427-8 (PMC10567849; doi:10.1007/s00277-023-05427-8)
Supplement: Supplementary file 1 — ESM 1 (DOCX 74.7 KB) [file 277_2023_5427_MOESM1_ESM.docx]

**Impact of residual tumor cells in the stem cell collection on multiple myeloma patients receiving autologous stem cell transplantation**

*Jingyu Xu^1,2^, Wenqiang Yan^1,2^, Huishou Fan^1,2^, Jiahui Liu^1,2^, Lingna Li^1,2^, Chenxing Du^1,2^, Shuhui Deng^1,2^, Weiwei Sui^1,2^, Yan Xu^1,2^, Lugui Qiu^1,2^, Gang An^1,2^ **

*1 State Key Laboratory of Experimental Hematology, National Clinical Research Center for Blood Diseases, Haihe Laboratory of Cell Ecosystem, Institute of Hematology & Blood Diseases Hospital, Chinese Academy of Medical Sciences & Peking Union Medical College, Tianjin 300020, China*

*2 Tianjin Institutes of Health Science, Tianjin 301600, China*

***Correspondence**: Gang An, 288 Nanjing Road, Tianjin, 300020, P.R.China,

E-mail: angang@ihcams.ac.cn, Tel: 86-13502181109

**Supplementary Information**

**Supplementary Table 1. Baseline characteristics of the entire cohort (n=89)**

| **Characteristics** | **No. of Patients (N=89, %)** |
| --- | --- |
| Age (years) | 54 [37-69] |
| Sex |  |
| Male | 56 (62.9) |
| Female | 33 (37.1) |
| Immunoglobulin subtype |  |
| IgG | 42 (47.3) |
| IgA | 26 (29.2) |
| Light chain | 10 (11.2) |
| IgD | 5 (5.6) |
| Non-secretory | 6 (6.7) |
| DS stage |  |
| I | 1 (1.1) |
| II | 8 (9.0) |
| III | 79 (88.8) |
| Missing | 1 (1.1) |
| ISS stage |  |
| I | 11 (12.4) |
| II | 42 (47.2) |
| III | 32 (36.0) |
| Missing | 4 (4.4) |
| R-ISS stage |  |
| I | 7 (7.9) |
| II | 55 (61.8) |
| III | 21 (23.6) |
| Missing | 6 (6.7) |
| Cytogenetic risk |  |
| Standard risk | 50 (56.1) |
| High risk | 24 (27.0) |
| Missing | 15 (16.9) |
| Laboratory values at diagnosis (range) |  |
| Hb (g/L) | 95 [55-162] |
| Serum albumin (g/L) | 34.3 [18.5-49.2] |
| Serum β2-MG (mg/L) | 4.43 [1.57-44.1] |
| Serum creatinine (μmol/L) | 74.7 [43-490.8] |
| Serum calcium (mmol/L) | 2.3 [1.86-4.01] |
| LDH | 167.4 [46.5-620.3] |
| MRD of the BM before ASCT (1×10^-5^) |  |
| Negative | 28 (31.5) |
| Positive | 61 (68.5) |
| MRD of the SCC before ASCT (1×10^-5^) |  |
| Negative | 68 (76.4) |
| Positive | 21 (23.6) |

Values are presented as median [range] or n (%).

DS, Durie-Salmon; ISS, International Staging System; R-ISS, Revised International Staging System; Hb, hemoglobin; β2-MG, β2-microglobulin; LDH, lactate dehydrogenase; MRD, minimal residual disease; BM, bone marrow; ASCT, autologous stem cell transplantation; SCC, stem cell collection.

**Supplementary Table 2. MRD status in the BM and SCC**

| **Variables** | **MRD+ in the BM** | **MRD+ in the SCC** | **P value** |
| --- | --- | --- | --- |
| Sensitivity |  |  | P<0.001 |
| 10^−6^ | 62 (69.6) | 22 (24.4) |  |
| 10^−5^ | 61 (68.5) | 21 (23.3) |  |
| 10^−4^ | 51 (57.3) | 11 (12.2) |  |
| Numeric level of tumor cells in the samples |  |  | P=0.019 |
| 10^1^-10^2^ | 30/62 (48.4) | 17/22 (77.3) |  |
| 10^3^-10^5^ | 32/62 (51.6) | 5/22 (22.7) |  |

Values are presented as n (%).

MRD, minimal residual disease; SCC, stem cell collection; BM, bone marrow.

**Supplementary Table 3. Impact of plerixafor on MRD status in the SCC**

|  | **Plerixafor** | **No plerixafor** | **P value** |
| --- | --- | --- | --- |
| No. of patients | 33 | 56 |  |
| No. of patients with MRD-positive SCC | 8/33 (24.2) | 13/56 (23.2) | 0.912 |
| Percentage of tumor cells (%) | 0.008 (0.001-0.1292) | 0.01 (0.0007-0.069) | 0.920 |
| Numeric level of tumor cells in the SCC |  |  | NA |
| 10^1^ | 1 | 0 |  |
| 10^2^ | 4 | 11 |  |
| 10^3^ | 2 | 2 |  |
| 10^4^ | 1 | 0 |  |

MRD, minimal residual disease; SCC, stem cell collection.

**Supplementary Table 4. Post-ASCT responses of patients with PR after induction**

| **Post-induction response** | **Post-ASCT response** | | **Best response** | |
| --- | --- | --- | --- | --- |
|  | **≥VGPR** | **≥CR** | **≥VGPR** | **≥CR** |
| PR+ | 40.0% | 10% | 60% | 30% |
| PR- | 43.7% | 12.5% | 66.6% | 20% |

ASCT, autologous stem cell transplantation; PR, partial response; VGPR, very good partial response; CR, complete response.


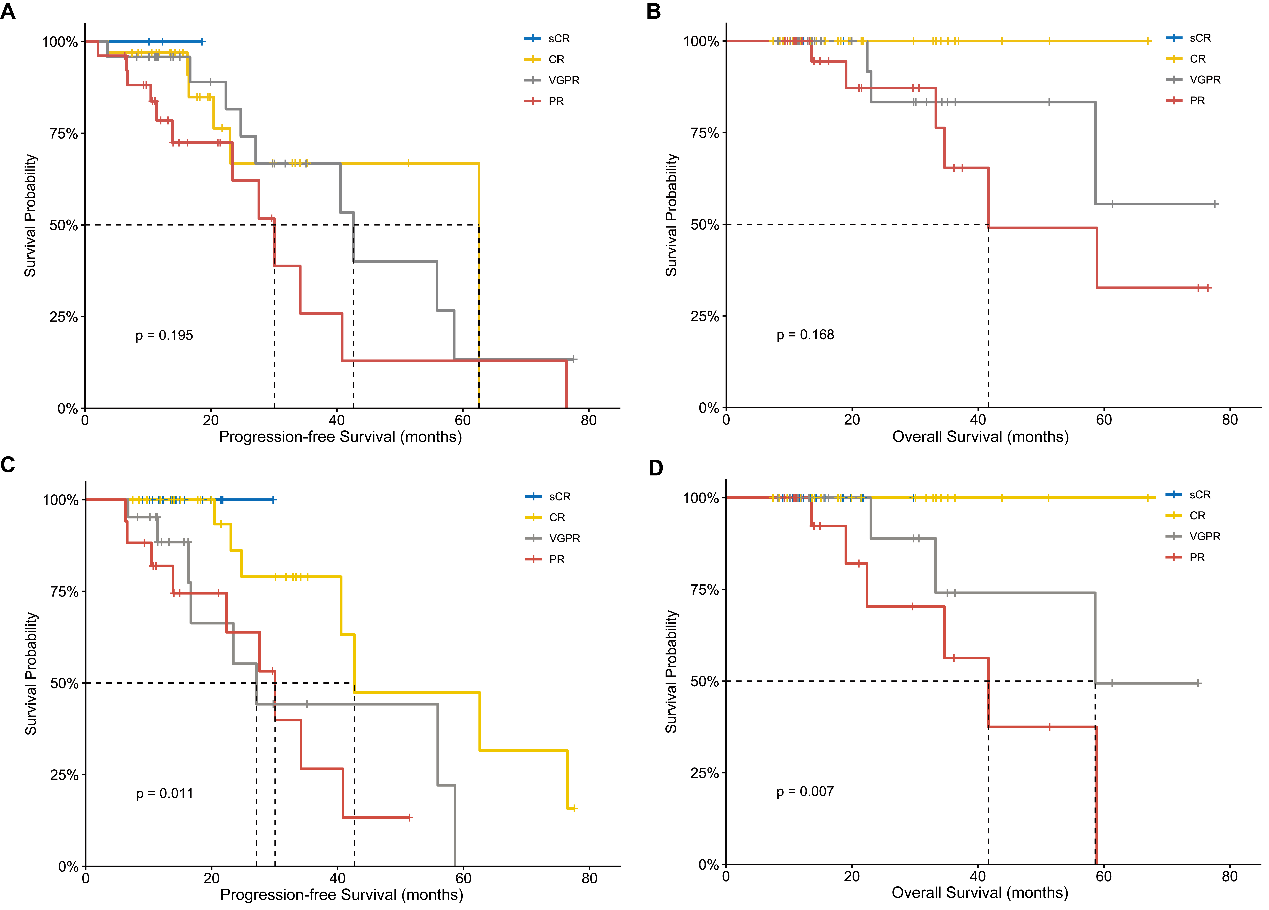


**Supplementary Figure 1.** **Kaplan–Meier survival curves for patients with multiple myeloma according to the response before (A, B) and after (C, D) ASCT.** ASCT, autologous stem cell transplantation; VGPR, very good partial response; CR, complete response; PR, partial response; sCR: stringent complete response.
